# Crystal structure and Hirshfeld surface analysis of *N*-(2,6-di­methyl­phen­yl)-2-[3-hy­droxy-2-oxo-3-(2-oxoprop­yl)indolin-1-yl]acetamide

**DOI:** 10.1107/S2056989022007848

**Published:** 2022-08-18

**Authors:** Intissar Nchioua, Abdulsalam Alsubari, Joel T. Mague, Youssef Ramli

**Affiliations:** a Laboratory of Medicinal Chemistry, Drug Sciences Research Center, Faculty of Medicine and Pharmacy, Mohammed V University in Rabat, Morocco; b Laboratory of Medicinal Chemistry, Faculty of Clinical Pharmacy, 21 September University, Yemen; c Department of Chemistry, 8 Tulane University, New Orleans, LA 70118, USA; Venezuelan Institute of Scientific Research, Venezuela

**Keywords:** crystal structure, hydrogen bond, indole, aryl­acetamide, Hirshfeld surface

## Abstract

The cup-shaped conformation of the title mol­ecule, C_21_H_22_N_2_O_4_, is largely determined by an intra­molecular N—H⋯O hydrogen bond. In the crystal, double layers of mol­ecules are formed by O—H⋯O and C—H⋯O hydrogen bonds. A Hirshfeld surface analysis was performed, which confirms the regions that are active for inter­molecular inter­actions.

## Abstract 2

The cup-shaped conformation of the title mol­ecule is largely determined by an intra­molecular N—H⋯O hydrogen bond. In the crystal, double layers of mol­ecules are formed by O—H⋯O and C—H⋯O hydrogen bonds.

## 1. Chemical context

**Figure 1:**
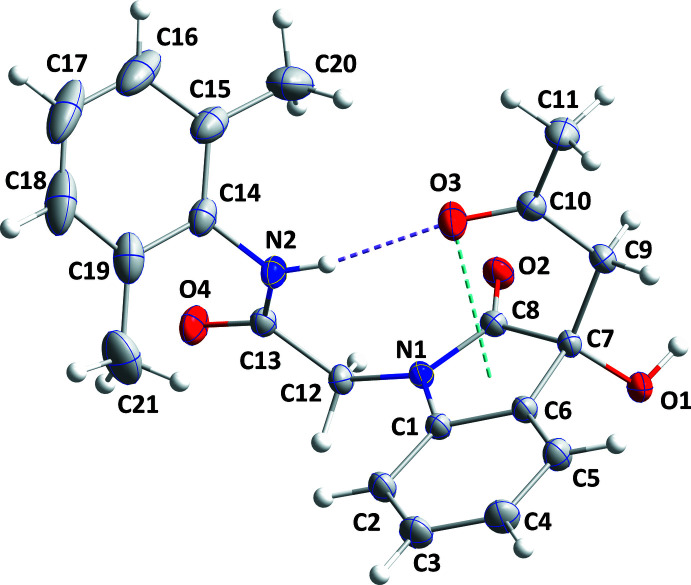
The title mol­ecule with labeling scheme and 50% probability ellipsoids. The intra­molecular N—H⋯O hydrogen bond and C=O⋯ring inter­action are depicted, respectively by violet and light-blue dashed lines.

1*H*-Indole-2,3-dione, also known as isatin, represents a synthetically useful substrate that can be used to prepare a broad range of heterocyclic compounds, including examples of pharmacological significance (Bekircan & Bektas, 2008 ▸). Its derivates are biologically active and have significant importance in medicinal chemistry (Feng *et al.*, 2010 ▸). They show potent anti­convulsant activity at low concentrations (Mathur & Nain, 2014 ▸), as well as anti­bacterial (Hu *et al.*, 2017 ▸), anti­cancer (Ding *et al.*, 2020 ▸) and anti­tubercular (Nath *et al.*, 2020 ▸) activities. Aryl­acetamide-based compounds have attracted increasing attention because of their important pharmacological activities (Beccalli *et al.*, 2007 ▸; Valeur & Bradley, 2009 ▸; Allen & Williams, 2011 ▸; Missioui *et al.*, 2021 ▸, 2022*a* ▸,*b* ▸,*c* ▸). As part of our inter­est in the identification of bioactive compounds, we report herein on the synthesis, crystal structure and Hirshfeld surface analysis of the title aryl­acetamide-based derivative containing an isatin moiety, namely *N*-(2,6-di­methyl­phen­yl)-2-[3-hy­droxy-2-oxo-3-(2-oxoprop­yl)indolin-1-yl]acetamide (Fig. 1 ▸) 
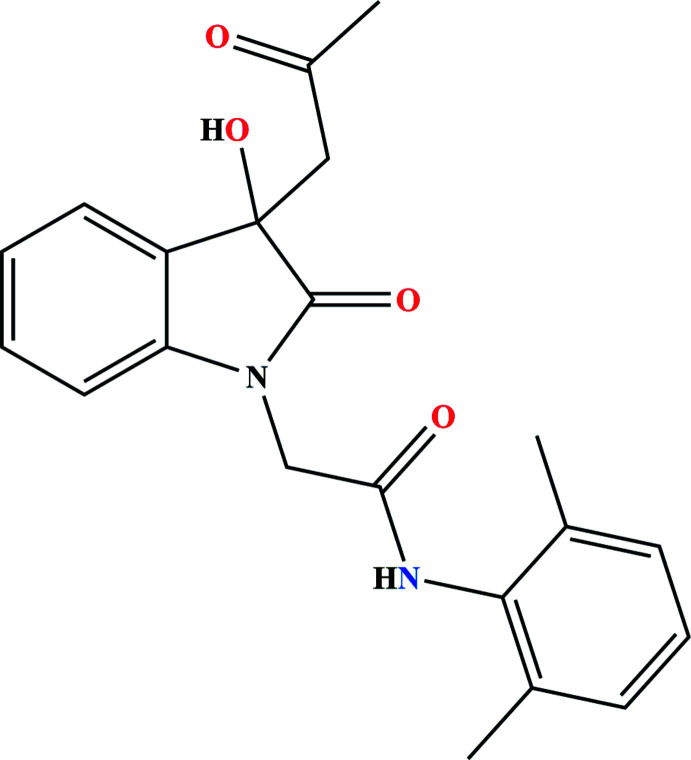


## 2. Structural commentary

The mol­ecule adopts a cup-shaped conformation (Fig. 1 ▸), which is largely determined by the intra­molecular N2—H2*A*⋯O3 hydrogen bond (Table 1 ▸). As this places O3 directly over the five-membered ring [O3⋯centroid = 2.7062 (8) Å, C10⋯centroid = 2.9956 (9) Å, C10=O3⋯centroid = 99.56 (9)°], there is the possibility of an added C=O⋯π inter­action reinforcing the observed conformation. The indole moiety is slightly non-planar as seen from the 1.89 (3)° dihedral angle between the mean planes of its constituent rings. The dihedral angle between the mean plane of the C1/C6/C7/C8/N1 ring and that of the C12/C13/N2/O4 unit is 82.83 (5)° while that between the latter plane and the mean plane of the C14–C19 ring is 72.24 (4)°. All bond distances and bond angles appear as expected for the given formulation.

## 3. Supra­molecular features

**Figure 2:**
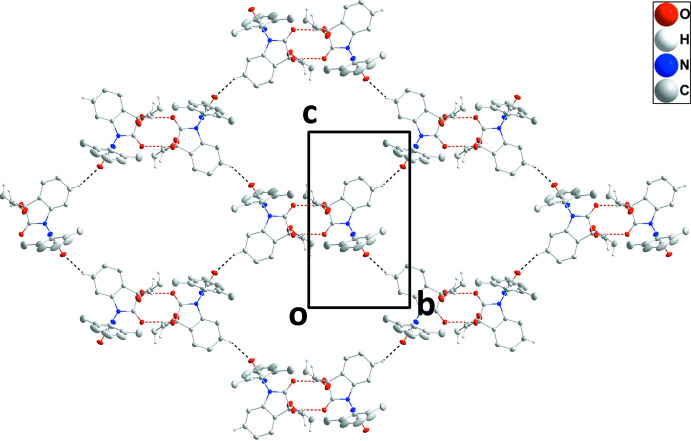
A plan view of a portion of one layer viewed along the *a*-axis direction. O—H⋯O and C—H⋯O hydrogen bonds are depicted, respectively, by red and black dashed lines while intra­molecular inter­actions and non-inter­acting hydrogen atoms are omitted for clarity.

**Figure 3:**
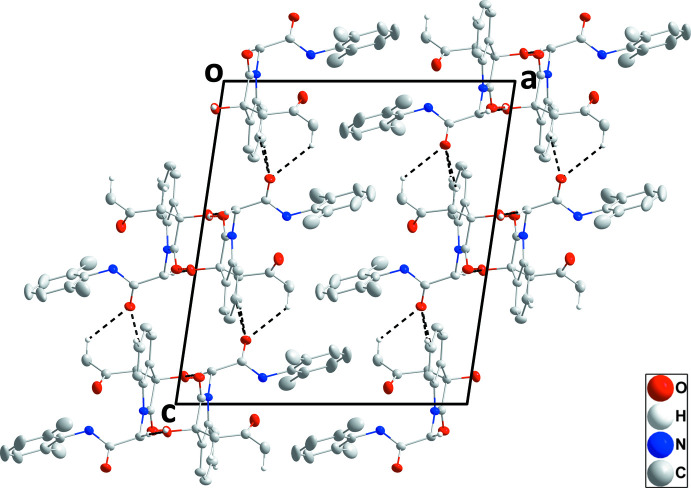
Packing viewed along the *b*-axis direction with O—H⋯O and C—H⋯O hydrogen bonds depicted, respectively, by red and black dashed lines. Intra­molecular inter­actions and non-inter­acting hydrogen atoms are omitted for clarity.

In the crystal, centrosymmetric dimers are formed by self-complementary O1—H1⋯O2 hydrogen bonds (Table 1 ▸) and these units are assembled into corrugated layers parallel to the *bc* plane by C3—H3⋯O4 hydrogen bonds (Table 1 ▸ and Fig. 2 ▸). Although these layers clearly contain large pores, they are combined in pairs across centers of symmetry by C9—H9*A*⋯O4, C11—H11*B*⋯O4 and C12—H12*A*⋯O1 hydrogen bonds (Table 1 ▸) so that the pores in one layer are capped by mol­ecules in the second and the resulting double layer has no significant pores (Fig. 3 ▸).

## 4. Database survey

A search of the Cambridge Structural Database (CSD version 5.43 updated to March 2022; Groom *et al.*, 2016 ▸) with the fragment A provided 28 hits, most of which contained a benzyl group attached to the ring nitro­gen atom. Of these, seven [DEVVUY (Liu *et al.*, 2018 ▸), DIDVAO (Makaev *et al.*, 2006 ▸), ODUWIV (Duan *et al.*, 2013 ▸), PUZBAQ (Becerra *et al.*, 2020 ▸), PUZBEU (Becerra *et al.*, 2020 ▸), PUZBIY (Becerra *et al.*, 2020 ▸) and PUZBOE (Becerra *et al.*, 2020 ▸)] are most similar to the title mol­ecule having a β-carbonyl group in the substituent attached to the saturated carbon of the five-membered ring. As in the title compound, all of these form dimers through complementary O— H⋯O hydrogen bonds between the hy­droxy and keto groups and these units are also further assembled into chains and/or layers by hydrogen-bonding inter­actions. 
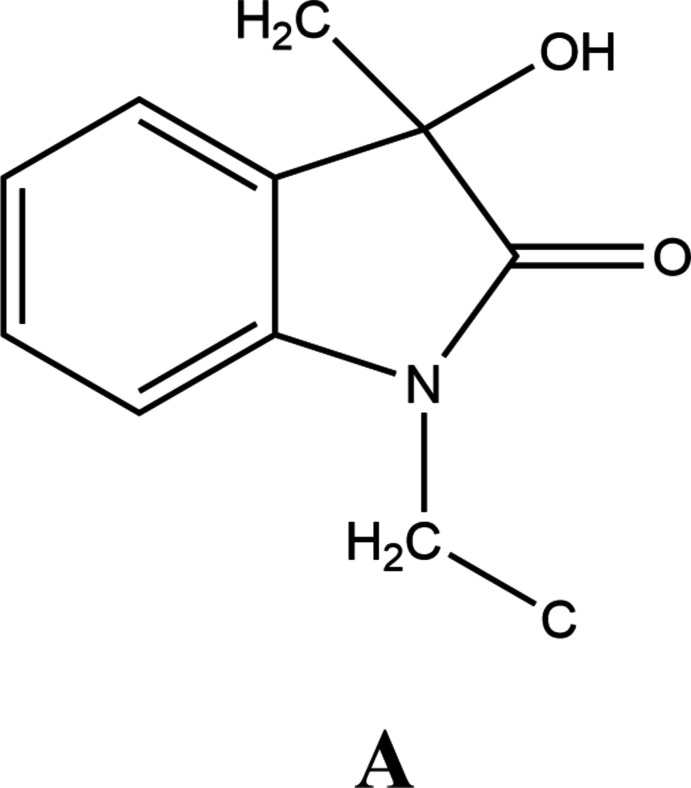


## 5. Hirshfeld surface analysis

**Figure 4:**
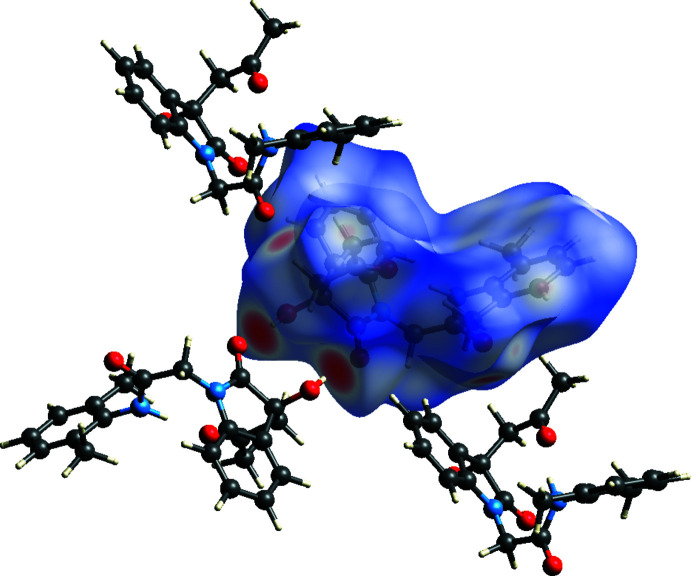
The Hirshfeld surface for the title mol­ecule with three close neighbors added.

**Figure 5:**
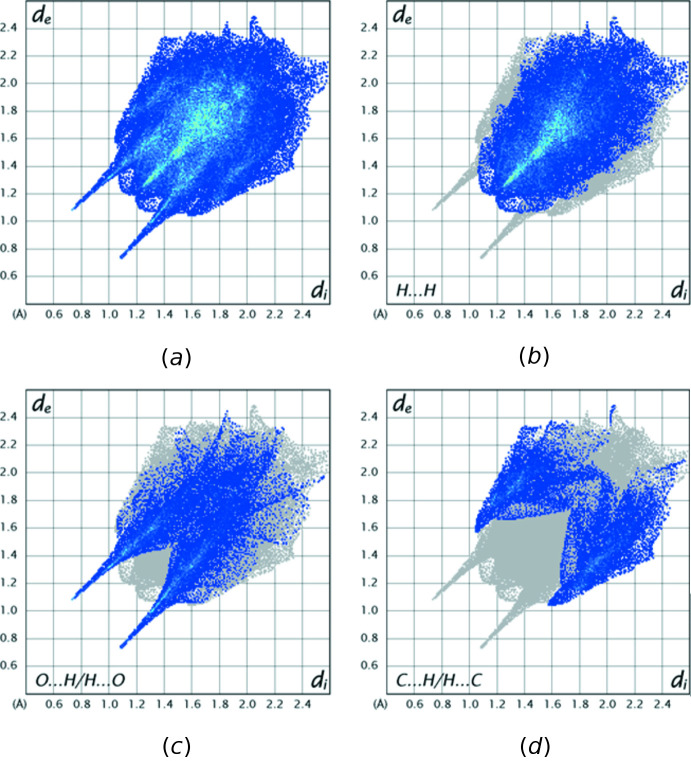
Fingerprint plots for the title mol­ecule: (*a*) all contacts, (*b*) H⋯H contacts, (*c*) O⋯H/H⋯O contacts and (*d*) C⋯H/H⋯C contacts.

The analysis was performed with *CrystalExplorer 21.5* (Spackman *et al.*, 2021 ▸) with the details of the pictorial output described in a recent publication (Tan *et al.*, 2019 ▸). Fig. 4 ▸ shows the *d*_norm_ surface for the asymmetric unit plotted over the limits −0.6060 to 1.5193 a.u. together with three adjacent mol­ecules that are hydrogen-bonded to it. The one on the lower left, adjacent to the pair of intense red spots, is the second half of one inversion dimer with these red spots indicating the strong O1—H1⋯O2 hydrogen bonds (*cf*. Fig. 2 ▸). The mol­ecules above and below the surface are members of two adjacent layers of mol­ecules (*cf*. Fig. 3 ▸), which are linked by the C9—H9*A*⋯O4 hydrogen bonds (lighter red spots). Fig. 5 ▸ *a* presents a fingerprint plot of all inter­molecular inter­actions while Fig. 5 ▸ *b* shows the 55.2% of these attributable to H⋯H inter­actions. Fig. 5 ▸ *c* and 5*d* delineate the O⋯H/H⋯O (24.1%) and C⋯H/H⋯C (17.8%) inter­actions, respectively.

## 6. Synthesis and crystallization

Indoline-2,3-dione (0.1g, 0.0679 mmol) was taken up in 10 mL of acetone under stirring. Solid potassium carbonate (0.11 g, 0.815 mmol) was added in one portion. Then, the dark-colored suspension was raised to room temperature and stirred for a further 1 h. The appropriate 2-chloro-*N*-(2,6-di­methyl­phen­yl)acetamide (0.119 g, 0.0679 mmol) and potassium iodide (0.05 g, 0.301 mmol) were added. Then, the reaction mixture was stirred at 353.15–373 K for 2 h until the reaction was complete, which was confirmed using TLC (ethyl acetate:hexane, 40:60). The resulting solid was filtered and recrystallized from ethanol to give title compound as colorless crystals. Yield: 64%; m.p. 527.15–529.15 K. FT–IR (ATR, υ, cm^−1^) 3292 υ (N—H amide), 1021 υ (N—C amide), 1675 υ (C=O amide), 1708 υ (C=O lactam), 1615 υ (C=O ketone), 3073 υ(C—H_arom_), 1175 υ(C—N), 2952 υ(C—H, CH_3_), 3348 (O—H). ^1^H NMR (DMSO–*d*_6_) δ ppm: 9.086 (*s*, 1H, NH); 7.011–7.338 (*m*, 7H, H_arom_); 6.134 (*s*, 1H, OH); 3.16-4.52 (2d, 2H, CH_2_); 2.03 (*s*, 6H, 2 CH_3_) 1.97 (*s*, 3H, CH_3_). ^13^C NMR (DMSO–*d*_6_) δ ppm: 207.448 (C=O), 177.126 (C=O_lactam_), 166.770 (C=O_amide_), 143.329; 135.794; 134.718; 131.196; 129.746; 128.268; 127.327; 124.150; 123.094; 109.196 (12CH_arom_), 72.740 (Cq), 51.075 (CH_2_—N), 40.200 (CH_2_—COCH_3_), 31.024 (CH_3_), 18.498 (2 CH_3_). Its mass spectrum showed a mol­ecular ion peak (*M*H^+^, *m*/*z* = 367.15799 and *M*Na^+^, *m*/*z* = 389.13943) that conforms to its mol­ecular formula C_21_H_22_N_2_O_4_

## 7. Refinement

Crystal data, data collection and structure refinement details are summarized in Table 2 ▸. Hydrogen atoms attached to carbon were included as riding contributions in idealized positions (C—H = 0.95–0.99 Å) with isotropic displacement parameters tied to those of the attached atoms [*U*_iso_(H) = 1.2–1.5*U*_eq_(C)]. Those attached to nitro­gen and to oxygen were placed in locations derived from a difference map and refined with DFIX 0.91 0.01 and DFIX 0.84 0.01 instructions, respectively.

## Supplementary Material

Crystal structure: contains datablock(s) global, I. DOI: 10.1107/S2056989022007848/zn2021sup1.cif

Structure factors: contains datablock(s) I. DOI: 10.1107/S2056989022007848/zn2021Isup2.hkl

Supporting information file. DOI: 10.1107/S2056989022007848/zn2021Isup3.cml

CCDC reference: 2194736

Additional supporting information:  crystallographic information; 3D view; checkCIF report

## Figures and Tables

**Table 1:** Hydrogen-bond geometry (Å, °)

*D*—H⋯*A*	*D*—H	H⋯*A*	*D*⋯*A*	*D*—H⋯*A*
O1—H1⋯O2^i^	0.864 (15)	1.942 (15)	2.7829 (9)	164.1 (14)
N2—H2*A*⋯O3	0.874 (15)	2.154 (15)	3.0193 (10)	170.3 (13)
C3—H3⋯O4^ii^	0.95	2.44	3.3280 (12)	155
C9—H9*A*⋯O4^iii^	0.99	2.33	3.2537 (11)	154
C11—H11*B*⋯O4^iii^	0.98	2.59	3.2988 (12)	129
C12—H12*A*⋯O1^iv^	0.99	2.60	3.5835 (11)	173

Symmetry codes: (i) 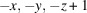 ; (ii) 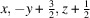 ; (iii) 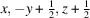 ; (iv) 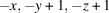 .

**Table 2:** Experimental details

Crystal data	
Chemical formula	C_21_H_22_N_2_O_4_
*M*_r_	366.40
Crystal system, space group	Monoclinic, *P*2_1_/*c*
Temperature (K)	150
*a*, *b*, *c* (Å)	13.8608 (5), 8.8352 (3), 15.5411 (6)
β (°)	98.468 (1)
*V* (Å^3^)	1882.46 (12)
*Z*	4
Radiation type	Mo *K*α
μ (mm^−1^)	0.09
Crystal size (mm)	0.46 × 0.37 × 0.26

Data collection	
Diffractometer	Bruker D8 QUEST PHOTON 3
Absorption correction	Numerical (*SADABS*; Krause *et al.*, 2015 ▸)
*T*_min_, *T*_max_	0.95, 0.98
No. of measured, independent and observed [*I* > 2σ(*I*)] reflections	101980, 6815, 5846
*R*_int_	0.035
(sin θ/λ)_max_ (Å^−1^)	0.759

Refinement	
*R*[*F* ^2^ > 2σ(*F* ^2^)], *wR*(*F* ^2^), *S*	0.045, 0.129, 1.07
No. of reflections	6815
No. of parameters	254
H-atom treatment	H atoms treated by a mixture of independent and constrained refinement
Δρ_max_, Δρ_min_ (e Å^−3^)	0.42, −0.31

Computer programs: *APEX4* and *SAINT* (Bruker, 2021 ▸), *SHELXT* (Sheldrick, 2015*a* ▸), *SHELXL2018/1* (Sheldrick, 2015*b* ▸), *DIAMOND* (Brandenburg & Putz, 2012 ▸) and *SHELXTL* (Sheldrick, 2008 ▸).

## supplementary crystallographic information

### Crystal data

**Table:**

C_21_H_22_N_2_O_4_	*F*(000) = 776
*M**_r_* = 366.40	*D*_x_ = 1.293 Mg m^−^^3^
Monoclinic, *P*2_1_/*c*	Mo *K*α radiation, λ = 0.71073 Å
*a* = 13.8608 (5) Å	Cell parameters from 9714 reflections
*b* = 8.8352 (3) Å	θ = 3.0–32.6°
*c* = 15.5411 (6) Å	µ = 0.09 mm^−^^1^
β = 98.468 (1)°	*T* = 150 K
*V* = 1882.46 (12) Å^3^	Block, colourless
*Z* = 4	0.46 × 0.37 × 0.26 mm

### Data collection

**Table:**

Bruker D8 QUEST PHOTON 3 diffractometer	6815 independent reflections
Radiation source: fine-focus sealed tube	5846 reflections with *I* > 2σ(*I*)
Graphite monochromator	*R*_int_ = 0.035
Detector resolution: 7.3910 pixels mm^-1^	θ_max_ = 32.6°, θ_min_ = 3.0°
φ and ω scans	*h* = −21→21
Absorption correction: numerical (*SADABS*; Krause *et al.*, 2015)	*k* = −13→13
*T*_min_ = 0.95, *T*_max_ = 0.98	*l* = −23→23
101980 measured reflections	

### Refinement

**Table:**

Refinement on *F*^2^	Primary atom site location: dual
Least-squares matrix: full	Secondary atom site location: difference Fourier map
*R*[*F*^2^ > 2σ(*F*^2^)] = 0.045	Hydrogen site location: mixed
*wR*(*F*^2^) = 0.129	H atoms treated by a mixture of independent and constrained refinement
*S* = 1.07	*w* = 1/[σ^2^(*F*_o_^2^) + (0.0697*P*)^2^ + 0.4122*P*] where *P* = (*F*_o_^2^ + 2*F*_c_^2^)/3
6815 reflections	(Δ/σ)_max_ = 0.001
254 parameters	Δρ_max_ = 0.42 e Å^−^^3^
0 restraints	Δρ_min_ = −0.31 e Å^−^^3^

### Special details

**Table:**

Experimental. The diffraction data were obtained from 9 sets of frames, each of width 0.5° in ω or φ, collected with scan parameters determined by the "strategy" routine in *APEX3*. The scan time was 5 sec/frame.
Geometry. All esds (except the esd in the dihedral angle between two l.s. planes) are estimated using the full covariance matrix. The cell esds are taken into account individually in the estimation of esds in distances, angles and torsion angles; correlations between esds in cell parameters are only used when they are defined by crystal symmetry. An approximate (isotropic) treatment of cell esds is used for estimating esds involving l.s. planes.
Refinement. Refinement of F^2^ against ALL reflections. The weighted R-factor wR and goodness of fit S are based on F^2^, conventional R-factors R are based on F, with F set to zero for negative F^2^. The threshold expression of F^2^ > 2sigma(F^2^) is used only for calculating R-factors(gt) etc. and is not relevant to the choice of reflections for refinement. R-factors based on F^2^ are statistically about twice as large as those based on F, and R- factors based on ALL data will be even larger. H-atoms attached to carbon were placed in calculated positions (C—H = 0.95 - 0.99 Å) and were included as riding contributions with isotropic displacement parameters 1.2 - 1.5 times those of the attached atoms. Those attached to nitrogen and to oxygen were placed in locations derived from a difference map and refined with DFIX 0.91 0.01 and DFIX 0.84 0.01 instructions, respectively.

### Fractional atomic coordinates and isotropic or equivalent isotropic displacement parameters (Å^2^)

**Table:**

	*x*	*y*	*z*	*U*_iso_*/*U*_eq_	
O1	−0.01619 (5)	0.15240 (7)	0.58519 (4)	0.02331 (13)	
H1	−0.0211 (11)	0.0551 (17)	0.5810 (10)	0.035*	
O2	0.06628 (5)	0.15275 (7)	0.41710 (4)	0.02433 (13)	
O3	0.27892 (5)	0.18850 (8)	0.55047 (5)	0.03248 (16)	
O4	0.20771 (5)	0.56774 (9)	0.30204 (4)	0.02884 (15)	
N1	0.10667 (5)	0.38567 (7)	0.47826 (4)	0.01780 (13)	
N2	0.28454 (5)	0.43751 (9)	0.41823 (5)	0.02231 (14)	
H2A	0.2780 (10)	0.3724 (17)	0.4593 (10)	0.035 (3)*	
C1	0.12285 (6)	0.44754 (8)	0.56329 (5)	0.01798 (14)	
C2	0.14520 (7)	0.59580 (9)	0.58717 (6)	0.02279 (16)	
H2	0.154105	0.670797	0.545243	0.027*	
C3	0.15409 (7)	0.63026 (10)	0.67608 (6)	0.02693 (17)	
H3	0.169109	0.730952	0.694915	0.032*	
C4	0.14134 (7)	0.51983 (11)	0.73723 (6)	0.02757 (18)	
H4	0.147813	0.545934	0.797102	0.033*	
C5	0.11902 (7)	0.37041 (10)	0.71126 (5)	0.02376 (16)	
H5	0.110095	0.294882	0.752878	0.029*	
C6	0.11028 (6)	0.33536 (9)	0.62380 (5)	0.01846 (14)	
C7	0.08199 (6)	0.18914 (8)	0.57660 (5)	0.01790 (14)	
C8	0.08473 (6)	0.23521 (9)	0.48075 (5)	0.01807 (14)	
C9	0.15003 (6)	0.05606 (9)	0.60396 (5)	0.02081 (15)	
H9A	0.147274	0.032787	0.665866	0.025*	
H9B	0.125732	−0.033851	0.569376	0.025*	
C10	0.25502 (6)	0.08274 (10)	0.59283 (6)	0.02231 (15)	
C11	0.32861 (7)	−0.02778 (12)	0.63580 (7)	0.0316 (2)	
H11A	0.307193	−0.131044	0.619844	0.047*	
H11B	0.335063	−0.015875	0.699085	0.047*	
H11C	0.391777	−0.008787	0.616620	0.047*	
C12	0.10666 (6)	0.47028 (9)	0.39869 (5)	0.01999 (14)	
H12A	0.077300	0.570684	0.406183	0.024*	
H12B	0.063673	0.417265	0.351674	0.024*	
C13	0.20497 (6)	0.49478 (9)	0.36878 (5)	0.01973 (14)	
C14	0.37969 (6)	0.45788 (11)	0.39438 (6)	0.02663 (18)	
C15	0.42552 (8)	0.33352 (14)	0.36257 (7)	0.0365 (2)	
C16	0.51776 (9)	0.3568 (2)	0.33836 (10)	0.0557 (4)	
H16	0.550264	0.275060	0.315182	0.067*	
C17	0.56220 (9)	0.4971 (2)	0.34769 (11)	0.0664 (5)	
H17	0.625396	0.510315	0.332070	0.080*	
C18	0.51564 (10)	0.6172 (2)	0.37936 (10)	0.0579 (4)	
H18	0.547197	0.712839	0.385442	0.069*	
C19	0.42263 (8)	0.60162 (14)	0.40288 (7)	0.0381 (2)	
C20	0.37803 (12)	0.18097 (16)	0.35535 (11)	0.0515 (3)	
H20A	0.315758	0.187197	0.316412	0.077*	
H20B	0.366401	0.147447	0.413048	0.077*	
H20C	0.420847	0.108401	0.331858	0.077*	
C21	0.37171 (12)	0.73438 (15)	0.43604 (10)	0.0528 (3)	
H21A	0.322214	0.772932	0.389673	0.079*	
H21B	0.419331	0.814269	0.454501	0.079*	
H21C	0.340299	0.702735	0.485625	0.079*	

### Atomic displacement parameters (Å^2^)

**Table:**

	*U*^11^	*U*^22^	*U*^33^	*U*^12^	*U*^13^	*U*^23^
O1	0.0214 (3)	0.0181 (3)	0.0320 (3)	−0.0025 (2)	0.0089 (2)	0.0019 (2)
O2	0.0324 (3)	0.0196 (3)	0.0212 (3)	−0.0036 (2)	0.0045 (2)	−0.0041 (2)
O3	0.0261 (3)	0.0303 (3)	0.0419 (4)	−0.0001 (3)	0.0079 (3)	0.0139 (3)
O4	0.0303 (3)	0.0361 (4)	0.0206 (3)	0.0004 (3)	0.0053 (2)	0.0107 (2)
N1	0.0230 (3)	0.0147 (3)	0.0162 (3)	−0.0012 (2)	0.0045 (2)	0.0007 (2)
N2	0.0221 (3)	0.0242 (3)	0.0213 (3)	0.0006 (2)	0.0051 (2)	0.0069 (2)
C1	0.0214 (3)	0.0153 (3)	0.0178 (3)	−0.0010 (2)	0.0045 (2)	−0.0007 (2)
C2	0.0296 (4)	0.0157 (3)	0.0237 (3)	−0.0032 (3)	0.0062 (3)	−0.0013 (3)
C3	0.0348 (4)	0.0198 (4)	0.0266 (4)	−0.0037 (3)	0.0057 (3)	−0.0062 (3)
C4	0.0356 (5)	0.0269 (4)	0.0203 (3)	−0.0020 (3)	0.0044 (3)	−0.0053 (3)
C5	0.0311 (4)	0.0226 (4)	0.0179 (3)	−0.0012 (3)	0.0049 (3)	0.0008 (3)
C6	0.0226 (3)	0.0153 (3)	0.0179 (3)	−0.0010 (2)	0.0044 (2)	−0.0002 (2)
C7	0.0206 (3)	0.0145 (3)	0.0193 (3)	−0.0019 (2)	0.0050 (2)	0.0012 (2)
C8	0.0196 (3)	0.0155 (3)	0.0193 (3)	−0.0005 (2)	0.0037 (2)	−0.0005 (2)
C9	0.0235 (3)	0.0157 (3)	0.0236 (3)	0.0004 (3)	0.0045 (3)	0.0029 (3)
C10	0.0236 (3)	0.0200 (3)	0.0236 (3)	0.0015 (3)	0.0044 (3)	0.0010 (3)
C11	0.0271 (4)	0.0310 (4)	0.0374 (5)	0.0081 (3)	0.0072 (3)	0.0093 (4)
C12	0.0228 (3)	0.0196 (3)	0.0180 (3)	0.0010 (3)	0.0043 (3)	0.0041 (2)
C13	0.0237 (3)	0.0186 (3)	0.0173 (3)	−0.0003 (3)	0.0044 (3)	0.0011 (2)
C14	0.0215 (3)	0.0354 (5)	0.0230 (4)	−0.0008 (3)	0.0034 (3)	0.0095 (3)
C15	0.0299 (4)	0.0478 (6)	0.0334 (5)	0.0128 (4)	0.0102 (4)	0.0136 (4)
C16	0.0342 (5)	0.0853 (11)	0.0517 (7)	0.0270 (6)	0.0199 (5)	0.0313 (7)
C17	0.0237 (5)	0.1103 (14)	0.0668 (9)	0.0030 (7)	0.0118 (5)	0.0503 (10)
C18	0.0323 (5)	0.0797 (10)	0.0591 (8)	−0.0212 (6)	−0.0020 (5)	0.0332 (8)
C19	0.0328 (5)	0.0454 (6)	0.0342 (5)	−0.0131 (4)	−0.0010 (4)	0.0135 (4)
C20	0.0586 (8)	0.0398 (6)	0.0598 (8)	0.0159 (6)	0.0215 (7)	−0.0014 (6)
C21	0.0691 (9)	0.0350 (6)	0.0522 (7)	−0.0196 (6)	0.0019 (6)	−0.0015 (5)

### Geometric parameters (Å, º)

**Table:**

O1—C7	1.4242 (10)	C9—H9B	0.9900
O1—H1	0.864 (15)	C10—C11	1.4965 (13)
O2—C8	1.2248 (9)	C11—H11A	0.9800
O3—C10	1.2167 (11)	C11—H11B	0.9800
O4—C13	1.2266 (10)	C11—H11C	0.9800
N1—C8	1.3655 (10)	C12—C13	1.5187 (11)
N1—C1	1.4170 (10)	C12—H12A	0.9900
N1—C12	1.4450 (10)	C12—H12B	0.9900
N2—C13	1.3467 (11)	C14—C15	1.3956 (15)
N2—C14	1.4329 (11)	C14—C19	1.4005 (15)
N2—H2A	0.874 (15)	C15—C16	1.4002 (16)
C1—C2	1.3840 (11)	C15—C20	1.497 (2)
C1—C6	1.3948 (11)	C16—C17	1.382 (3)
C2—C3	1.4026 (12)	C16—H16	0.9500
C2—H2	0.9500	C17—C18	1.370 (3)
C3—C4	1.3913 (13)	C17—H17	0.9500
C3—H3	0.9500	C18—C19	1.3976 (17)
C4—C5	1.4017 (13)	C18—H18	0.9500
C4—H4	0.9500	C19—C21	1.499 (2)
C5—C6	1.3818 (11)	C20—H20A	0.9800
C5—H5	0.9500	C20—H20B	0.9800
C6—C7	1.5087 (11)	C20—H20C	0.9800
C7—C9	1.5279 (11)	C21—H21A	0.9800
C7—C8	1.5501 (11)	C21—H21B	0.9800
C9—C10	1.5091 (12)	C21—H21C	0.9800
C9—H9A	0.9900		
			
C7—O1—H1	106.6 (10)	C10—C11—H11B	109.5
C8—N1—C1	110.76 (6)	H11A—C11—H11B	109.5
C8—N1—C12	123.77 (7)	C10—C11—H11C	109.5
C1—N1—C12	125.35 (6)	H11A—C11—H11C	109.5
C13—N2—C14	120.85 (7)	H11B—C11—H11C	109.5
C13—N2—H2A	120.0 (9)	N1—C12—C13	116.66 (7)
C14—N2—H2A	118.0 (9)	N1—C12—H12A	108.1
C2—C1—C6	122.48 (7)	C13—C12—H12A	108.1
C2—C1—N1	127.85 (7)	N1—C12—H12B	108.1
C6—C1—N1	109.65 (6)	C13—C12—H12B	108.1
C1—C2—C3	116.96 (8)	H12A—C12—H12B	107.3
C1—C2—H2	121.5	O4—C13—N2	123.73 (8)
C3—C2—H2	121.5	O4—C13—C12	118.34 (7)
C4—C3—C2	121.27 (8)	N2—C13—C12	117.91 (7)
C4—C3—H3	119.4	C15—C14—C19	122.55 (10)
C2—C3—H3	119.4	C15—C14—N2	118.49 (9)
C3—C4—C5	120.58 (8)	C19—C14—N2	118.96 (9)
C3—C4—H4	119.7	C14—C15—C16	117.43 (13)
C5—C4—H4	119.7	C14—C15—C20	121.16 (10)
C6—C5—C4	118.55 (8)	C16—C15—C20	121.41 (12)
C6—C5—H5	120.7	C17—C16—C15	120.95 (14)
C4—C5—H5	120.7	C17—C16—H16	119.5
C5—C6—C1	120.16 (7)	C15—C16—H16	119.5
C5—C6—C7	130.47 (7)	C18—C17—C16	120.38 (11)
C1—C6—C7	109.27 (7)	C18—C17—H17	119.8
O1—C7—C6	109.46 (6)	C16—C17—H17	119.8
O1—C7—C9	110.96 (6)	C17—C18—C19	121.27 (14)
C6—C7—C9	114.71 (7)	C17—C18—H18	119.4
O1—C7—C8	107.99 (6)	C19—C18—H18	119.4
C6—C7—C8	101.59 (6)	C18—C19—C14	117.39 (13)
C9—C7—C8	111.59 (6)	C18—C19—C21	120.81 (13)
O2—C8—N1	125.24 (7)	C14—C19—C21	121.80 (10)
O2—C8—C7	126.03 (7)	C15—C20—H20A	109.5
N1—C8—C7	108.66 (6)	C15—C20—H20B	109.5
C10—C9—C7	114.46 (6)	H20A—C20—H20B	109.5
C10—C9—H9A	108.6	C15—C20—H20C	109.5
C7—C9—H9A	108.6	H20A—C20—H20C	109.5
C10—C9—H9B	108.6	H20B—C20—H20C	109.5
C7—C9—H9B	108.6	C19—C21—H21A	109.5
H9A—C9—H9B	107.6	C19—C21—H21B	109.5
O3—C10—C11	121.37 (8)	H21A—C21—H21B	109.5
O3—C10—C9	121.73 (8)	C19—C21—H21C	109.5
C11—C10—C9	116.90 (7)	H21A—C21—H21C	109.5
C10—C11—H11A	109.5	H21B—C21—H21C	109.5
			
C8—N1—C1—C2	−178.96 (8)	C6—C7—C8—N1	−2.55 (8)
C12—N1—C1—C2	−2.79 (13)	C9—C7—C8—N1	−125.24 (7)
C8—N1—C1—C6	−0.65 (9)	O1—C7—C9—C10	177.23 (7)
C12—N1—C1—C6	175.53 (7)	C6—C7—C9—C10	−58.09 (9)
C6—C1—C2—C3	−0.55 (13)	C8—C7—C9—C10	56.75 (9)
N1—C1—C2—C3	177.57 (8)	C7—C9—C10—O3	−13.37 (12)
C1—C2—C3—C4	0.25 (14)	C7—C9—C10—C11	167.08 (8)
C2—C3—C4—C5	−0.08 (15)	C8—N1—C12—C13	−98.64 (9)
C3—C4—C5—C6	0.18 (14)	C1—N1—C12—C13	85.66 (9)
C4—C5—C6—C1	−0.47 (13)	C14—N2—C13—O4	−0.69 (13)
C4—C5—C6—C7	−176.29 (8)	C14—N2—C13—C12	−179.30 (8)
C2—C1—C6—C5	0.68 (13)	N1—C12—C13—O4	−179.12 (8)
N1—C1—C6—C5	−177.74 (7)	N1—C12—C13—N2	−0.44 (11)
C2—C1—C6—C7	177.32 (7)	C13—N2—C14—C15	−107.45 (10)
N1—C1—C6—C7	−1.11 (9)	C13—N2—C14—C19	71.84 (12)
C5—C6—C7—O1	64.33 (11)	C19—C14—C15—C16	−0.29 (15)
C1—C6—C7—O1	−111.85 (7)	N2—C14—C15—C16	178.98 (9)
C5—C6—C7—C9	−61.13 (12)	C19—C14—C15—C20	179.24 (11)
C1—C6—C7—C9	122.69 (7)	N2—C14—C15—C20	−1.49 (15)
C5—C6—C7—C8	178.34 (9)	C14—C15—C16—C17	1.46 (18)
C1—C6—C7—C8	2.16 (8)	C20—C15—C16—C17	−178.06 (13)
C1—N1—C8—O2	179.22 (8)	C15—C16—C17—C18	−1.3 (2)
C12—N1—C8—O2	2.97 (12)	C16—C17—C18—C19	−0.1 (2)
C1—N1—C8—C7	2.07 (9)	C17—C18—C19—C14	1.18 (18)
C12—N1—C8—C7	−174.18 (7)	C17—C18—C19—C21	−178.97 (13)
O1—C7—C8—O2	−64.57 (10)	C15—C14—C19—C18	−1.01 (15)
C6—C7—C8—O2	−179.67 (8)	N2—C14—C19—C18	179.73 (10)
C9—C7—C8—O2	57.64 (10)	C15—C14—C19—C21	179.15 (11)
O1—C7—C8—N1	112.55 (7)	N2—C14—C19—C21	−0.12 (15)

### Hydrogen-bond geometry (Å, º)

**Table:**

*D*—H···*A*	*D*—H	H···*A*	*D*···*A*	*D*—H···*A*
O1—H1···O2^i^	0.864 (15)	1.942 (15)	2.7829 (9)	164.1 (14)
N2—H2*A*···O3	0.874 (15)	2.154 (15)	3.0193 (10)	170.3 (13)
C3—H3···O4^ii^	0.95	2.44	3.3280 (12)	155
C9—H9*A*···O4^iii^	0.99	2.33	3.2537 (11)	154
C11—H11*B*···O4^iii^	0.98	2.59	3.2988 (12)	129
C12—H12*A*···O1^iv^	0.99	2.60	3.5835 (11)	173

Symmetry codes: (i) −*x*, −*y*, −*z*+1; (ii) *x*, −*y*+3/2, *z*+1/2; (iii) *x*, −*y*+1/2, *z*+1/2; (iv) −*x*, −*y*+1, −*z*+1.
